# Survival outcomes post percutaneous coronary intervention: Why the hype about stent type? Lessons from a healthcare system in India

**DOI:** 10.1371/journal.pone.0196830

**Published:** 2018-05-24

**Authors:** Bhanu Duggal, Jyothi Subramanian, Mona Duggal, Pushpendra Singh, Meeta Rajivlochan, Sujata Saunik, Koundinya Desiraju, Archana Avhad, Usha Ram, Sayan Sen, Anurag Agrawal

**Affiliations:** 1 Department of Cardiology, AIIMS, Rishikesh, India; 2 Department of Health and Family Welfare, Government of Maharashtra, Mumbai, India; 3 Department of Community Medicine, PGIMER, Chandigarh, India; 4 Indraprastha Institute of Information Technology, New Delhi, India; 5 Department of Health and Family Welfare, Government of Maharashtra, Mumbai, India; 6 Institute of Genomics and Integrative Biology, New Delhi, India; 7 International Institute of Population Sciences, Mumbai, India; 8 Department of Cardiology, Hammersmith Hospital, London, United Kingdom; University of Tampere, FINLAND

## Abstract

A prospective, multicenter study was initiated by the Government of Maharashtra, India, to determine predictors of long-term outcomes of percutaneous coronary intervention (PCI) for coronary artery disease, and to compare the effectiveness of drug-eluting stents (DESs) and bare-metal stents (BMSs) in patients undergoing PCI under government-funded insurance. The present analysis included 4595 patients managed between August 2012 and November 2016 at any of 110 participating centers. Using the classical multivariable regression and propensity-matching approach, we found age to be the most important predictor of 1-year mortality and target lesion revascularization at 1 year post-PCI. However, using machine learning methods to account for unmeasured confounders and bias in this large observational study, we determined total stent length and number of stents deployed as the most important predictors of 1-year survival, followed by age and employment status. The unadjusted death rates were 5.0% and 3.8% for the BMS and DES groups, respectively (p = 0.185, log-rank test). The rate of re-hospitalization (p<0.001) and recurrence of unstable angina (p = 0.08) was significantly lower for DESs than for BMSs. Increased use of DES after 2015 (following establishment of a price cap on DESs) was associated with a sharp decrease in adjusted hazard ratios of DESs versus BMSs (from 0.94 in 2013 to 0.58 in 2016), suggesting that high price was limiting DES use in some high-risk patients. Since stented length and stent number were the most important predictors of survival outcomes, adopting an ischemia-guided revascularization strategy is expected to help improve outcomes and reduce procedural costs. In the elderly, PCI should be reserved for cases where the benefits outweigh the higher risk of the procedure. As unemployed patients had poorer long-term outcomes, we expect that implementation of a post-PCI cardiovascular rehabilitation program may improve long-term outcomes.

## Introduction

Percutaneous coronary intervention (PCI) with stent implantation is one of the most widely used cardiovascular interventions for the treatment of coronary artery disease (CAD). PCI with implantation of drug-eluting stents (DESs) is a contemporary treatment strategy that has been under intense scrutiny for both clinical and economic reasons. In particular, use of DESs is associated with increased costs to the healthcare system [1–3]. The initial economic analysis justified use of DESs, proposing that the increased upfront costs would be compensated by savings due to decreased recurrence of cardiovascular events and hospitalizations. However these analyses were restricted to the highly controlled settings of randomized trials, where only a single stent was implanted per lesion.

Although randomized trials remain the gold standard for comparative effectiveness studies, such investigations do not provide the practical means to answer a wide range of research questions. Furthermore, the protocol-driven conditions that form the framework of randomized trials cannot be adhered to in contemporary clinical practice. Thus, it remains unknown whether the data supporting DES use can be generalized to real-life cardiology practice, especially in countries such as India.

In 2012, the Government of Maharashtra, one of the largest states in India, introduced a government-funded insurance scheme to provide patients from socio-economically disadvantaged groups with access to high-cost medical care [1, 4, 5]. However, the variability in healthcare costs and the especially high annual costs associated with the use of DESs prompted policy makers to initiate a prospective, multicenter, observational registry to compare the efficacy of DESs versus BMSs in terms of all-cause mortality and risk of re-hospitalization, repeat PCI, and angina recurrence within 1 year of the index procedure in patients undergoing PCI under this government-funded insurance policy. The factors affecting all-cause mortality and rate of repeat revascularization were also studied.

## Materials and methods

This prospective study included medical centers in each district of Maharashtra. Participating centers had adequate facilities to provide standardized cardiovascular care. Patients covered under the government-funded insurance scheme were free to present to any of the participating centers to receive treatment. The complete electronic medical records of the treated patients were uploaded in a centralized database maintained by the Department of Health and Family Welfare of the Government of Maharashtra.

### Study population and data collection

The study was approved by the Ethics Committee of Grant Medical College and Sir J. J. Group of Hospitals, Mumbai. The study enrolled adult patients (aged 18 years and above) undergoing PCI with stent implantation in one or more coronary arteries, under the government-funded insurance scheme, in one of the participating hospitals. The choice of stent and post-PCI medications was at the discretion of the treating interventional cardiologist.

Prior to the start of the study, the research coordinators responsible for data collection participated in a training session where the standardized forms for data collection and manual of operations were reviewed to ensure consistency in data collection practices(S1 File). The patients were followed-up over a period of 1 year. Telephone interviews with the patients were recorded by the research interviewers after obtaining informed consent from the patient. As this study involved a telephone survey (questionnaire attached as S2–S4 Files), permission to obtain only verbal informed consent over the telephone (rather than written consent) was granted by the Ethics Committee. The procedural details were obtained from the electronic database maintained by the Government of Maharashtra, and entered in standardized forms. Patients who did not receive a stent during the index PCI and patients who died during hospitalization for the index procedure were excluded from the analysis [6, 7].

The details recorded in the telephone interview forms and standardized forms for procedural information were later filled by data entry operators into a dedicated electronic case report form and transmitted via the Internet to a central database at a specialized data center. The database was regularly monitored for source data documentation and missing or questionable data. Completions or corrections were made where possible. Patients with incomplete records were excluded from the present study.

### Definitions

In accordance with the provisions of the Academic Research Consortium, we used all-cause mortality as the most unbiased indicator of death outcomes. All-cause mortality is commonly used to describe the outcomes of clinical trials and observational studies, even though it may be a less specific indicator than mortality from cardiac causes. In this study, all deaths were considered of cardiac origin unless an unequivocal non-cardiac cause could be established. Other outcomes included repeat revascularization and recurrent angina. Repeat revascularization was defined as any PCI or coronary artery bypass graft surgery during the follow-up period. Stable angina was defined as pain precipitated by exertion and relieved by rest or sublingual nitroglycerin, with no change in pattern or severity for 6 weeks. Unstable angina was defined as either pain presenting at rest, or exertional pain of at least class III in the Canadian Cardiovascular Society (CCS) grading system, which increased in severity at by least one CCS class in 2 months [8].

### Study end points

The two end points of interest were all-cause mortality within 1 year after the index procedure and the combined outcome of all-cause mortality or repeat PCI during the same time frame [9].

### Statistical analyses

Baseline sociodemographic, clinical, angiographic, and treatment-related characteristics were compared between the DES and BMS groups Data are expressed as mean (standard deviation). Continuous variables were compared using Student’s t test, while categorical variables were compared using χ^2^ tests. The software R version 3.4.1 (R Foundation for Statistical Computing, Vienna, Austria) was used for all analyses.

#### Multivariable regression and propensity-matched analysis

Event rates between the index procedure and the 1-year follow-up were estimated using the Kaplan-Meier method and compared using the log-rank statistic. To obtain adjusted risk estimates for 1-year events, two analytic approaches were applied, namely multivariable risk adjustment and propensity-matched analysis. The following covariates were included: location of hospital (within Mumbai or out of Mumbai), age at PCI, sex, employment status, education level, diabetes, hypertension, CAD history, year of PCI (2012, 2013, 2014, 2015, or 2016), minimum stent diameter (smallest value was retained in patients who received more than one stent during index PCI), total stented length (total length of all stents placed in the same lesion during index PCI), and total number of stents placed during index PCI.

For standard multivariable analysis, Cox proportional hazards regression was used to calculate adjusted hazard ratios (HRs) and associated confidence intervals (CIs) for the endpoints of interest while controlling for the above-listed covariates, with BMS data considered as reference. For the propensity-matched analysis, logistic regression was first used to develop a propensity score reflecting the probability of receiving a DES, conditional on the same previously listed covariates. Nearest-neighbor matching was then performed, wherein each patient in the DES group was matched with a patient in the BMS group who had an estimated logit score within 0.2 standard deviations of the score of the selected DES patient. The success of DES-to-BMS group matching was examined in terms of the weighted standardized differences in the distribution of baseline covariates. Finally, Cox proportional hazards regression was used to evaluate the risk of certain outcomes associated with DES use relative to the risk for the same outcome expected with BMS use. Furthermore, the multivariable model was used to predict the importance of each variable for survival outcomes. In the regression models, the coefficient of each variable reflects the impact of the variable on the outcome after adjusting for other predictors.

#### Random forest models

Because the coefficients in the multivariable regression models capture only the linear component of the association between each variable and the outcome, and our study included real-world data expected to exhibit nonlinear relationships, the importance of each variable was also assessed using random forest, a new machine learning algorithm providing an alternative approach to calculate propensity scores in such a way as to account for some degree of nonlinearity. Using random forests, we calculated propensity scores based on the same covariates mentioned above [10]. It is possible that the additional level of randomness implemented by the random forest classifier allowed less important variables to be expressed in predicting therapy exposure, thereby attenuating the magnitude of the effects. Subsequently, the propensity scores and covariates were included as predictors and random forest models were built to predict the outcomes. To make the prediction feasible and intuitive, we performed a series of simulations using data pertaining to 1000 patients. For each potential predictor:

1. Simulate the outcome in such a way that all variables other than the predictor of interest have the same value for all patients.
2. Predict outcome in these patients. At this stage, the difference in predictions is only due to the predictor of interest, since all other variables have the same value for all patients.
3. Repeat these steps 100 times using different constant values for variables other than the predictor of interest.

## Results

Between August 2012 and November 2016, we interviewed 4595 patients and their families. The patients had been treated at any of 110 participating centers across Maharashtra. Of the 4595 patients interviewed, 2202 received at least one BMS and were included in the BMS group. The remaining 2393 patients received only DESs and were included in the DES group (Table 1).

**Table 1:** Demographic and clinical characteristics of patients.

Variable	BMS group	DES group	p*
N	2202	2393	
Age, years, mean (SD)	57.79 (10.70)	55.98 (10.53)	<0.001
Sex, n (%)			0.042
Male	1583 (71.9)	1798 (75.1)	
Missing information	3 (0.1)	3 (0.1)	
Education level, n (%)			<0.001
None or up to primary school	1084 (49.2)	1003 (41.9)	
Middle school and above	1029 (46.7)	1323 (55.3)	
Missing information	89 (4.0)	67 (2.8)	
Employment status (%)			<0.001
Unemployed	1320 (59.9)	1342 (56.1)	
Employed	793 (36.0)	984 (41.1)	
Missing information	89 (4.0)	67 (2.8)	
Location of hospital, n (%)			0.001
Within Mumbai	629 (28.6)	784 (32.8)	
Out of Mumbai	1484 (67.4)	1543 (64.5)	
Missing information	89 (4.0)	66 (2.8)	
Diabetes, n (%)			0.002
No	1456 (66.1)	1518 (63.4)	
Yes	657 (29.8)	808 (33.8)	
Missing information	89 (4.0)	67 (2.8)	
Hypertension, n (%)			0.001
No	1260 (57.2)	1291 (53.9)	
Yes	853 (38.7)	1035 (43.3)	
Missing information	89 (4.0)	67 (2.8)	
Tobacco usage status, n (%)			<0.001
Current smoker	254 (11.5)	388 (16.2)	
Past smoker	294 (13.4)	343 (14.3)	
Non-smoker	1565 (71.1)	1595 (66.7)	
Missing information	89 (4.0)	67 (2.8)	
Medical history, n (%)			<0.001
Previous MI	310 (14.1)	479 (20.0)	
Acute coronary syndrome	1228 (55.8)	1147 (47.9)	
Chronic stable angina	635 (28.8)	747 (31.2)	
Positive stress test	9 (0.4)	10 (0.4)	
Missing information	20 (0.9)	10 (0.4)	
CAD history (%)			0.003
No	1844 (83.7)	1970 (82.3)	
Yes	269 (12.2)	356 (14.9)	
Missing information	89 (4.0)	67 (2.8)	
Aspirin use, n (%)			0.013
No	278 (12.6)	273 (11.4)	
Yes	1832 (83.2)	2053 (85.8)	
Missing information	92 (4.2)	67 (2.8)	
Clopidogrel use (%)			0.01
No	577 (26.2)	586 (24.5)	
Yes	1533 (69.6)	1740 (72.7)	
Missing information	92 (4.2)	67 (2.8)	
Prasugrel, n (%)			<0.001
No	1902 (86.4)	2021 (84.5)	
Yes	208 (9.4)	305 (12.7)	
Missing information	92 (4.2)	67 (2.8)	
Ticlopidine, n (%)			0.007
No	2100 (95.4)	2304 (96.3)	
Yes	10 (0.5)	22 (0.9)	
Missing information	92 (4.2)	67 (2.8)	
Dual antiplatelet therapy, n (%)			<0.001
No	479 (21.8)	428 (17.9)	
Yes	1631 (74.1)	1898 (79.3)	
Missing information	92 (4.2)	67 (2.8)	

Patients were stratified according to stent type. P-values were obtained using Fisher’s exact test or the chi-square test for categorical variables, and using the t-test for quantitative variables. BMS, bare-metal stent; CAD, coronary artery disease; DES, drug-eluting stent; MI, myocardial infarction; SD, standard deviation

Patients in the DES group were slightly younger, had higher education and employment levels, and were more likely to have undergone PCI at a hospital within Mumbai. The prevalence of diabetes, hypertension, family history of CAD, and smoking was higher in the DES group. Patients treated with DESs received longer stents and were more likely to have left anterior descending artery stenosis, as well as to receive dual antiplatelet therapy and newer antiplatelet agents. The covariate that was most different between the BMS and DES groups was the year in which PCI was done. (patients managed later were more often on DES).

A total of 2848 BMSs and 3338 DESs were deployed (Table 2 & Table 3), with some patients receiving multiple stents. The number of DESs increased significantly starting in 2015. There was no significant difference between the groups regarding the number of stents deployed per patient. DESs were, on average, significantly longer than BMSs.

**Table 2:** Stent details.

Variable	BMS group	DES group	P value
Year of PTCA, n (%)			<0.001
2012	543 (24.7)	216 (9.0)	
2013	790 (35.9)	469 (19.6)	
2014	300 (13.6)	396 (16.5)	
2015	364 (16.5)	746 (31.2)	
2016	205 (9.3)	566 (23.7)	
Total number of stents per patient, mean (SD)	1.55 (0.69)	1.53 (0.69)	0.616
Total stent length per patient, mean (SD)	33.43 (18.28)	36.57 (20.48)	<0.001
Maximum stent diameter, mean (SD)	3.09 (1.07)	3.06 (0.68)	0.276
Minimum stent diameter, mean (SD)	2.83 (0.45)	2.85 (0.42)	0.046

BMS, bare-metal stent; DES, drug-eluting stent; PTCA, percutaneous transluminal coronary angioplasty; SD, standard deviation

**Table 3:** Procedural details.

Location	BMS group	DES group	None
LAD stent, n (%)	1317 (28.7)	1739 (37.8)	1539 (33.5)
LCX stent, n (%)	646 (14.1)	634 (13.8)	3315 (72.1)
RCA stent, n (%)	841 (18.3)	919 (20.0)	2835 (61.7)

BMS, bare-metal stent; DES, drug-eluting stent; LAD, left anterior descending artery, LCX = left circumflex artery; RCA, right coronary artery; SD, standard deviation, None: Patient did not receive a stent in the said vessel in that patient.

**Fig 1:**
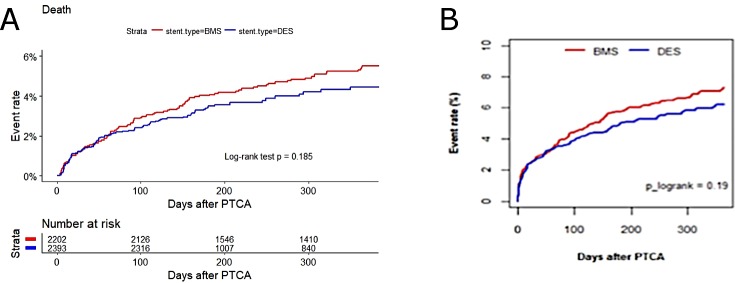
Univariate Kaplan-Meier curves for 1-year outcomes after PCI, stratified by stent type. (A) All-cause mortality. (B) Combined outcome of death or repeat PCI in the same vessel. BMS, bare-metal stent; DES, drug-eluting stent; PCI, percutaneous coronary intervention.

There were a total of 203 deaths within 1 year of the index procedure. Fig 1A shows the cumulative rates of post-PCI mortality over the course of 1 year, according to the type of stent received during the index PCI. The unadjusted death rates were 5.0% for the BMS group and 3.8% for the DES group (p = 0.185, log-rank test). With respect to the outcome of death and repeat PCI in the same vessel, there were a total of 243 such events within 1 year of the index procedure. Fig 1B shows the cumulative rates of combined outcomes at 1year post-PCI, according to the type of stent received during the index PCI. The unadjusted death rates were 5.5% for the BMS group and 4.3% for the DES group (p = 0.264, log-rank test). There was a significant difference between the two groups regarding the rate of re-hospitalization (p<0.001) and recurrent unstable angina (p = 0.006) over the course of the entire follow-up period (Table 4).

**Table 4:** Incidence of major outcomes after PCI with stent implantation.

Event	BMS group	DES group	p-value (chi-square test)
Re-hospitalization	104	69	<0.001
Repeat PCI	34	31	0.416
Advised repeat PCI	36	39	0.889
Unstable angina	163	137	0.008
Stable angina	131	161	0.298

BMS, bare-metal stent; DES, drug-eluting stent; PCI, percutaneous coronary intervention

### Predictors according to multivariable regression analysis

Patients with missing data regarding one or more covariates were excluded. A total of 4308 patients and 197 events (deaths) were included in the analysis of mortality outcomes; the multivariate Cox regression model revealed an adjusted HR for 1-year mortality of 0.83 (95% CI, 0.61–1.13) for DES use relative to BMS use. A total of 4300 patients and 217 events were included in the analysis of combined outcomes of mortality and repeat PCI in the same vessel; the multivariate Cox regression model revealed an adjusted HR for 1-year combined outcomes of 0.87 (95% CI, 0.65–1.16) for DES use relative to BMS use.

### Predictors following propensity-matching

**Fig 2:**
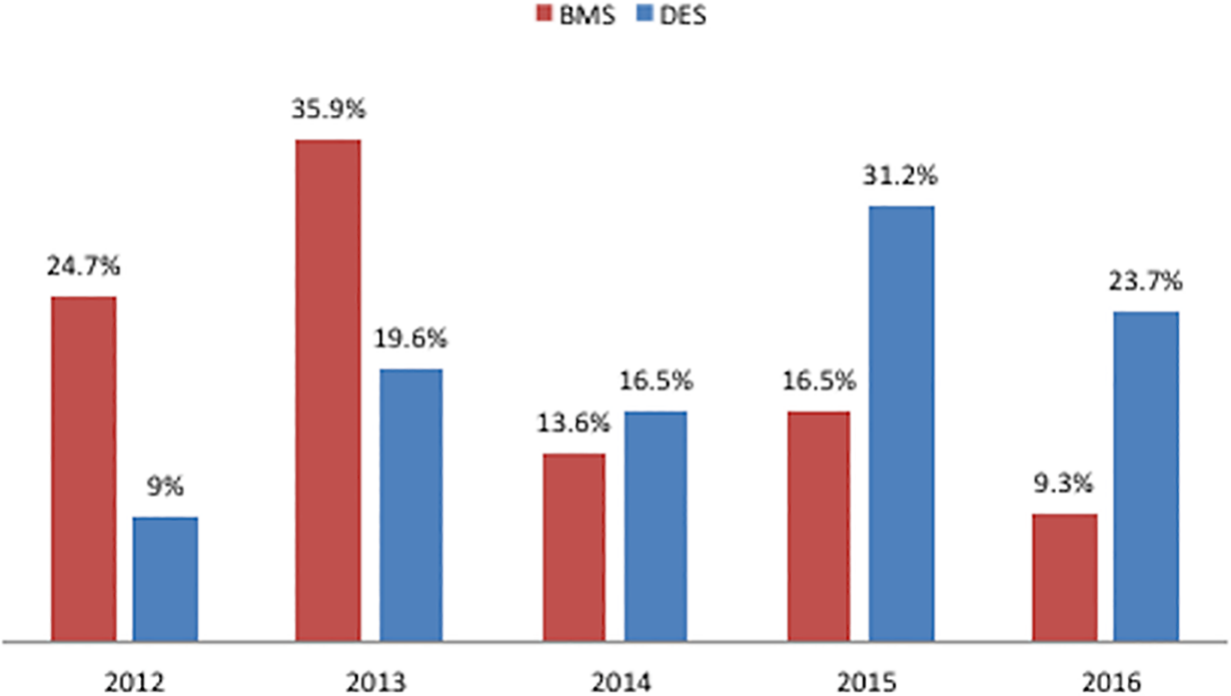
Absolute standardized differences in baseline characteristics, stratified according to stent type. (Left) Before matching. (Right) After matching. BMS, bare-metal stent; DES, drug-eluting stent.

**Fig 3:**
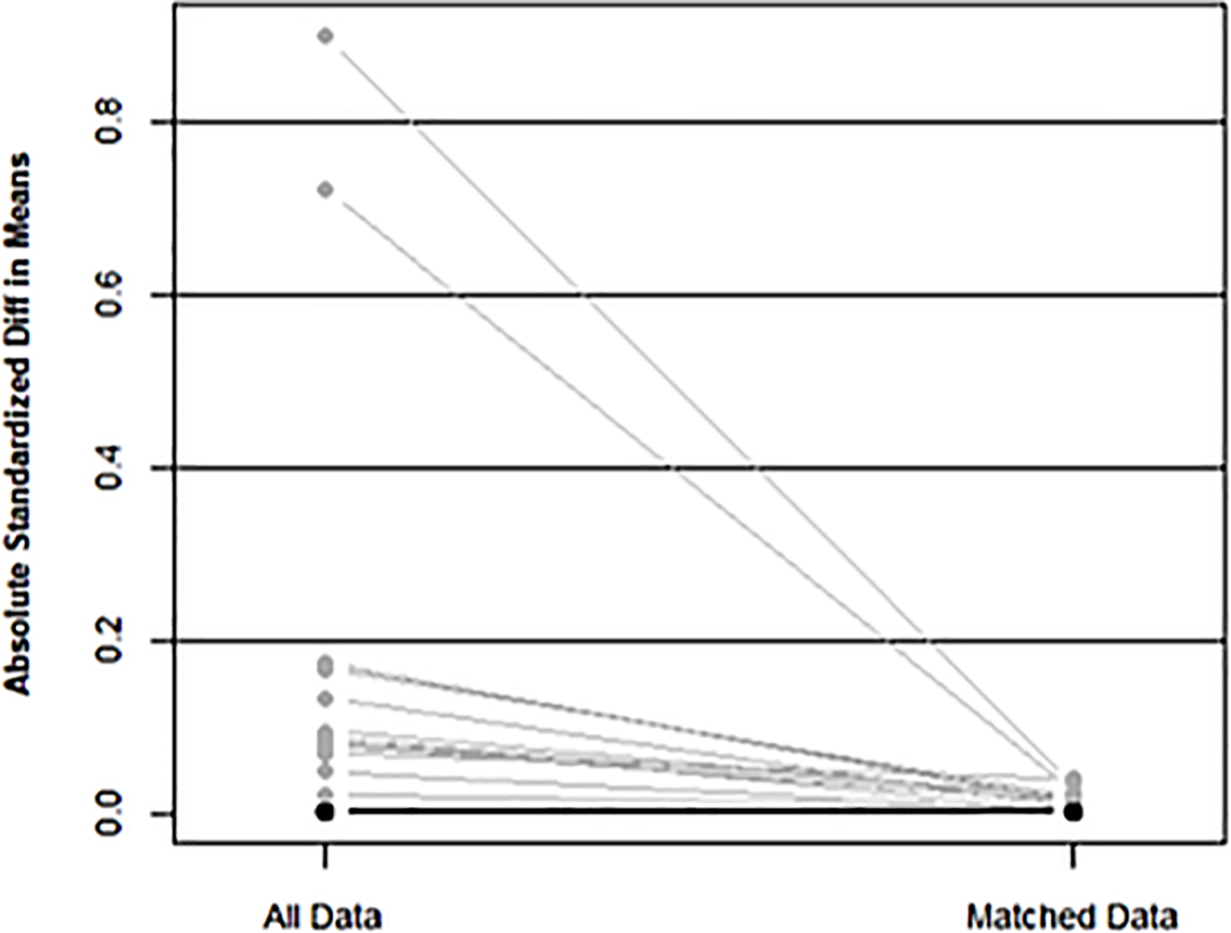
Temporal trend of stent usage by stent type. BMS, bare-metal stent; DES, drug-eluting stent.

Upon matching patients based on their propensity to receive DESs versus BMSs, we obtained a sub-cohort consisting of 1413 patients in each sub-group (Fig 2). The two sub-groups were highly similar (standardized mean difference, <10%) regarding baseline characteristics and overall logit score (Fig 3). The Cox regression analysis using the matched datasets revealed that, relative to BMS use, DES use had an HR of 0.90 (95% CI, 0.64–1.27) for 1-year mortality and 0.98 (95% CI, 0.71–1.36) for the combined outcome of mortality or repeat PCI within 1 year of the index procedure.

### DES versus BMS usage over the years

DES usage increased steadily over the years from 2012 to 2016 (Fig 3). Multivariable regression analysis for mortality outcomes was conducted separately for each year, and the adjusted HR for the propensity to receive a DES relative to the propensity to receive a BMS is summarized in Table 5 for each year of the study period. A sudden jump in the propensity to receive DESs occurred in 2015, when a price cap on DESs was established by law.

**Table 5:** Multivariable regression for propensity to receive a certain stent type.

Year of PTCA	Adjusted HR (95% CI), DES vs BMS use
**2012**	0.67 (0.29–1.53)
**2013**	0.94 (0.51–1.74)
**2014**	1.09 (0.52–2.32)
**2015**	0.71 (0.37–1.37)
**2016**	0.58 (0.28–1.18)

BMS, bare-metal stent; CI, confidence interval; DES, drug-eluting stent; HR, hazard ratio; PTCA, percutaneous transluminal coronary angioplasty

### Relative importance of predictor variables

**Fig 4:**
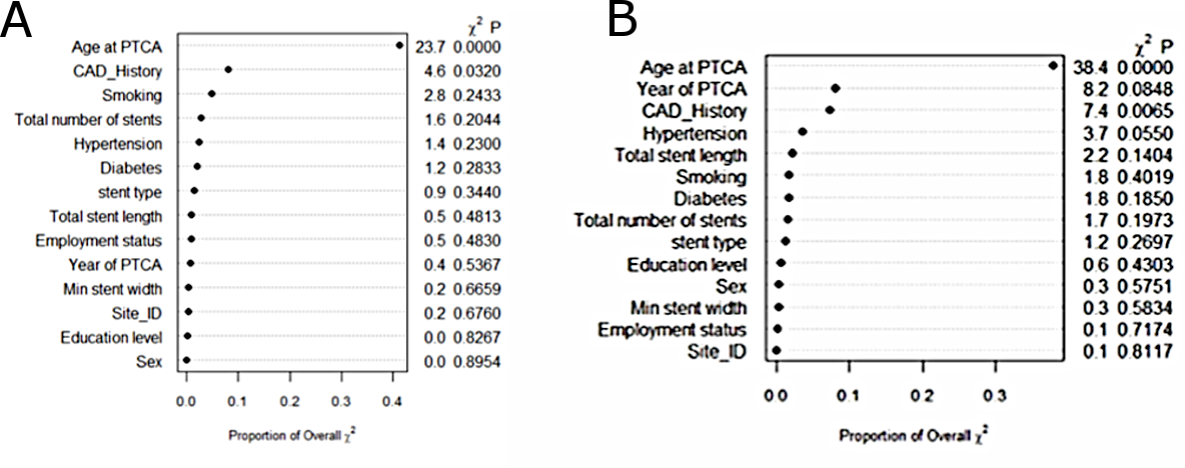
Estimated importance of predictors for 1-year outcomes after PCI. (A) All-cause mortality. (B) Combined outcome of death or repeat PCI in the same vessel. CAD, coronary artery disease; PTCA, percutaneous transluminal coronary angioplasty.

The proportion of explainable log-likelihood explained by each variable was considered to reflect the relative importance of each variable for predicting the outcomes of interest (all-cause death and death or repeat PCI within 1 year of the index procedure) using the multivariable regression model. The results are shown in Fig 4A (for all-cause death) and Fig 4B (for the combined outcome of death or repeat PCI). In this analysis, the top five most important predictors of all-cause mortality were age, hypertension, prior history of CAD, total stent length, and stent type; for the combined outcome of death or repeat PCI, the top five predictors were age, CAD history, smoking, total number of stents, and hypertension.

**Fig 5:**
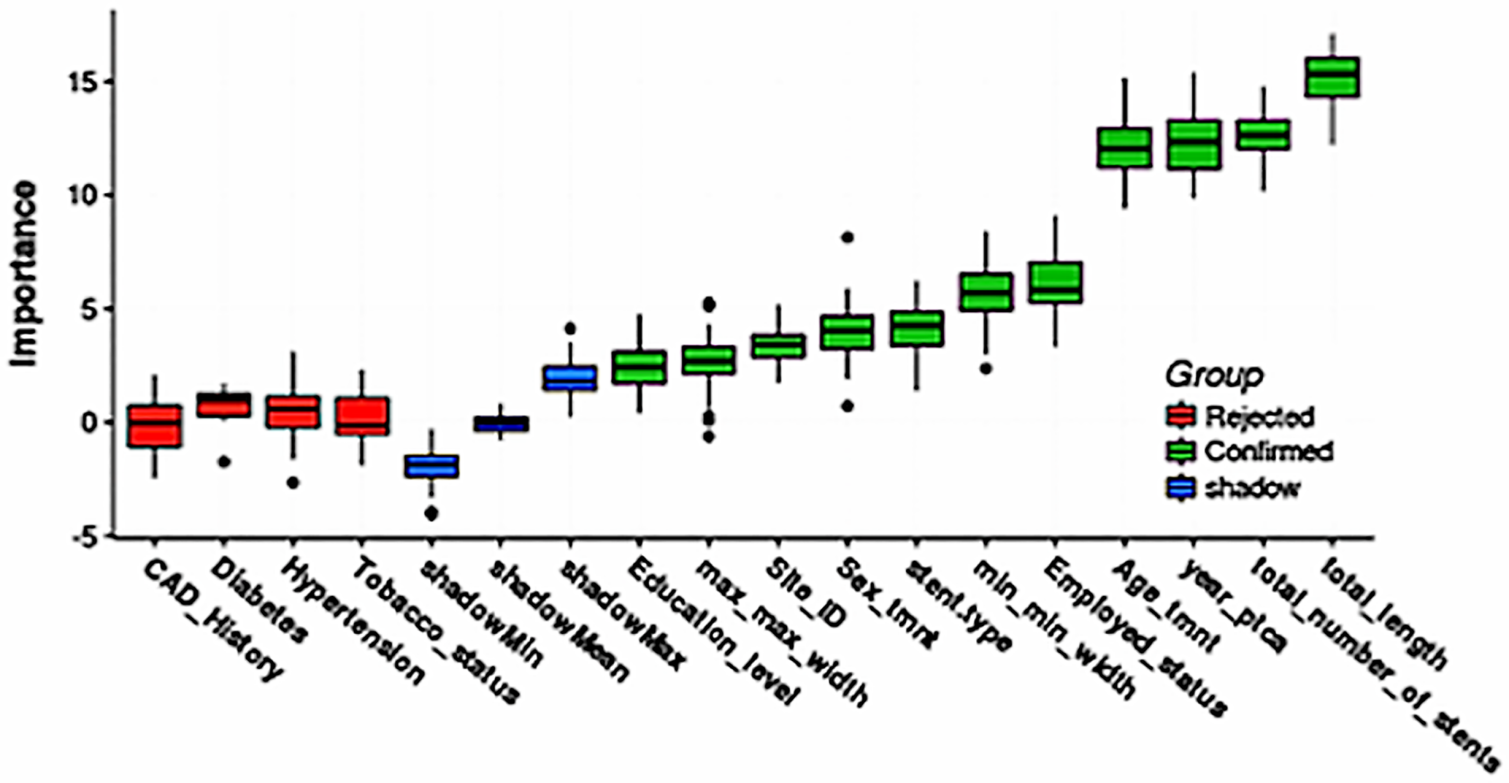
Random forest analysis of predictors of all-cause mortality at 1-year after post PCI. CAD, coronary artery disease; TMNT, treatment; PCI, percutaneous coronary intervention.

**Fig 6:**
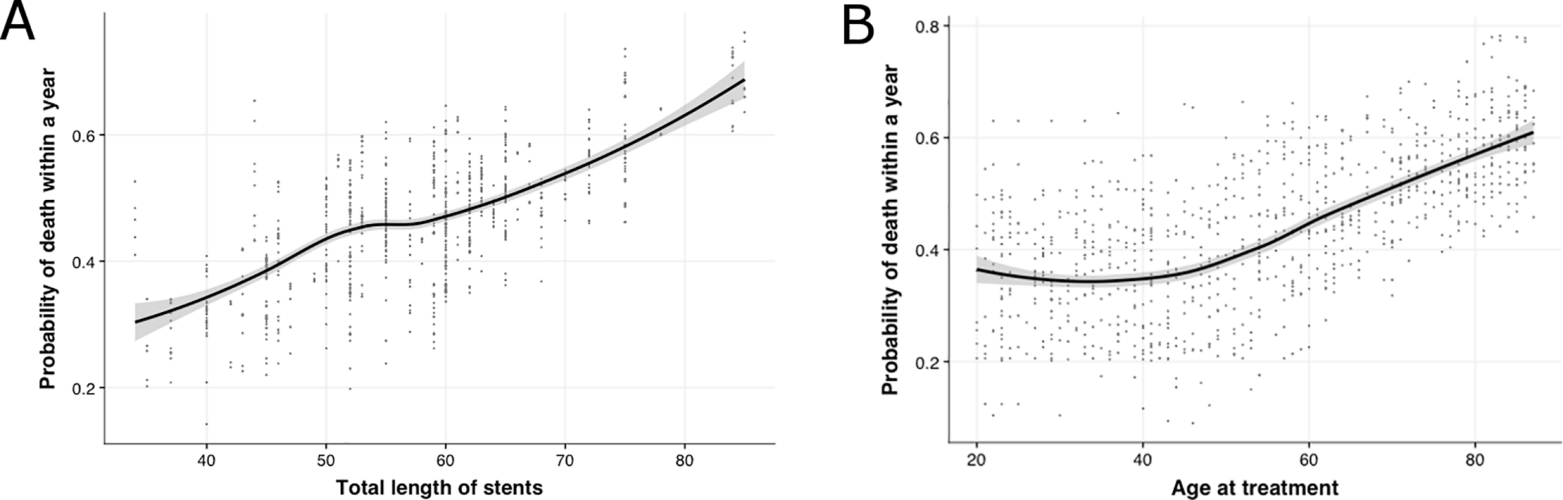
A. A linear trend was seen between the probability of death and stented length (i.e., total length of implanted stents) The risk of death increased significantly with total stented length (Fig 6A). After the age of 50 years, the risk of adverse outcomes increased significantly with age (Fig 6B). There was no difference in outcomes between BMSs and DESs wider than 4 mm (Fig 6C).

However, when using a random forest algorithm, which evaluates the importance of attributes relative to that of randomized (shadow) attributes (Fig 5), we found the total number of stents, total stented length, age at the time of index PCI, and employment status to be the most important predictors of 1-year mortality (Fig 6A & 6B).There was no difference in outcomes between BMSs and DESs wider than 4 mm.(Fig 6C). In the random forest model, which had an accuracy of about 70%, stented length and number of stents implanted were more important than stent type.

## Discussion

To our knowledge, this is the first study conducted in South Asia using the e-health records collected by the State Department of Health and Family Welfare. Our study brings novel insights that can be used to develop strategies for improving long-term survival outcomes, as well as to shape policy decisions regarding provision of insurance coverage to economically disadvantaged groups.

In our study, we found no difference in mortality according to stent type (DES versus BMS), but DES usage was associated with a lower rate of re-hospitalization and recurrent unstable angina. We feel that the most likely reason why these differences in recurrence rates did not translate into differences in repeat revascularization rate is that the patients could not afford a second procedure because their health insurance coverage had been exhausted. Often, this financial concern discourages and even prevents patients from visiting a cardiac care facility and rather motivates them to visit a nearby internist who would manage them conservatively and not indicate them for a repeat procedure [11, 12]. Indeed, there was a sudden decrease in HR of DES versus BMS use in 2015, the first year of follow-up of patients who had undergone PCI after regulation of stent prices by the Government of Maharashtra, which resulted in affordable DESs. The decrease in HR for DES versus BMS use seen in 2015 and 2016 would then be attributable to greater comparability of clinician-judged patient risks [13]. The higher costs of DESs have been justified by the decrease in restenosis rates and repeat revascularizations, especially in populations at high risk of restenosis after the initial PCI [14, 15].

Random forest analysis revealed that stent-related parameters (total stented length and number of stents, followed by stent diameter) were the most important factors associated with poor survival outcomes. Suh et al. found that a total stented length ≥31.5 mm was a predictor of stent thrombosis and mortality [16]. We found a linear association between stented length and 1-year mortality. [17, 18]. Our present finding is in agreement with previous observations that performing PCI of all angiographic stenoses, regardless of their ischemic potential, diminishes the benefit of relieving ischemia by exposing the patient to additional stent-related risks [19–21]. Though higher number of stents may indicate presence of multivessel and more severe disease, the decision to stent non-ischemic lesions provides no additional benefit over medical therapy. Ad-hoc PCI (concomitant coronary angiography and angioplasty) should be discouraged in multi-vessel disease, and a heart-team comprised of cardiovascular surgeons, interventional cardiologists, and primary cardiologists should be constituted in every hospital for optimal decision making [3, 20, 22–25]. Avoiding the implantation of additional stents reduces procedure and treatment costs at the outset, as well as in terms of better long-term outcomes.

On both Cox regression and logistic regression analyses, age emerged as one of the most important predictors of major adverse outcomes in this cohort. Though some recent studies reported improved survival rates in the elderly, this was not the case in our study, which used registry data [26, 27]. This discrepancy might be related to the composition of the cohort. Specifically, our cohort may have included patients with poorer physical status and more severe comorbidities. Both conventional and ensemble models revealed a linear increase in mortality outcomes with age. Thus, it might be prudent to preferentially indicate elderly patients for optimal medical management before considering PCI unless the survival benefit outweighs the procedure-related risk [28].

Employment status also emerged as an important factor for post-PCI outcomes. Specifically, unemployment was associated with poor outcomes, which is in agreement with previous observations [29]. Thus, in addition to adequate health insurance coverage, a program of cardiac rehabilitation encouraging patients to return to full-time work would likely help in improving long-term outcomes.

## Limitations

Part of the study was conducted in the form of a telephone survey, and response bias could not be excluded; however, we hope that the large size of the cohort has mitigated any discrepancies associated with response bias. Another potential limitation of the study is that we could not examine the effect of discontinuation of antiplatelet agents on the incidence of adverse outcomes; thus, we could not exclude the effect of the prolonged duration of dual antiplatelet therapy on major adverse cardiac events in patients who received DESs.

### What is already known

Compared to bare-metal stents, drug-eluting stents are associated with reduced mortality and repeat revascularization.

### What the study adds

We found that the total stented length and the number of stents were more important than the type of stent in terms of the rate of major adverse cardiac events, highlighting the need for strict adherence to the appropriate use criteria for stent procedures, and suggesting the possibility to rank healthcare providers according to the rate of AUC adoption.

## Supporting information

S1 File FORM O PLOS 1. (DOCX)

S2 File “SURVEY FORM PLOS 1. (DOC)

S3 File “HINDI1. (PDF)

S4 File “HINDI2. (PDF)

S5 File “MARATHI. (PDF)

S6 File Dataset used for analysis. (CSV)

S7 File Columns and their details. (PDF)
