# Supplementary material for: Survival outcomes post percutaneous coronary intervention: Why the hype about stent type? Lessons from a healthcare system in India
Source: PLoS One. 2018 May 24;13(5):e0196830. doi: 10.1371/journal.pone.0196830 (PMC5967815; doi:10.1371/journal.pone.0196830)
Supplement: S1 File — (DOCX) [file pone.0196830.s001.docx]

**O FORM**

**Case No**.

Age Gender:

**Date of Admission** Date of PTCA Date of Discharge

**Hospital where PTCA done**

**HISTORY**  Stemi (1) Unstable angina (2) Chronic Stable Angina (3) Positive stress test (4)

**Risk factors** : Htn (1) Diabetes (2) Family Hx (3) Smoking/Chewing(4) COPD (5) Past history of IHD (6)

**INVESTIGATIONS** Blood Pressure Blood Sugar LDL Cholesterol LVEF

TG Serum Creatinine HB Platelets

**ECG**---------------Acute MI (1), Acute ACS(2) ST-T changes(3) Non specific changes (4)

**TYPE OF VESSEL INVOLVED**............................LAD (1) LCX (2) RCA(3) Proximal LAD (4) Any other vessel --------

**Thrombus Seen**: Yes/No Thrombus Aspiration done . Yes/No

**NUMBER OF VESSELS INVOLVED**---------------ONE, TWO, THREE,

**PTCA**-----------------------------------LAD, LCX RCA,

**STENT IN LAD**-------------BMS /DES

No of stents ___________ Name of stent ________Size of stent WIdth............length............................

Size of distal stent Width........................... Lenth .................

Size of proximal stent Width........................... Lenth ...........

Stent at Site Width........................... Lenth ...........

**STENT IN LCX**----BMS/DES

No. of stents_____ ___ Name of stent _______________Size of stent Width................... Length ...........................

Size of distal stent Width........................... Lenth .................

Size of proximal stent Width........................... Lenth ...........

Stent at Site Width........................... Length ...........

**STENT IN RCA** BMS/DES

No .of stents_____ ___ Name of stent _________________________Size of stent ..Length............................

Size of distal stent Width........................... Length .................

Size of proximal stent Width........................... Lenth ...........

Stent at Site Width........................... Lenth ...........

**DRUGS**

ECOSPRINE DOSE CLOPIDOGREL DOSE OTHERS

PRASUGREL DOSE STATINS DOSE OHA

ACE-INHIBITORS DOSE BETA BLOCKERS DOSE

ANTIANGINAL- Antihypertensive

METFORMIN/GLIPTIN/PIO/INSULIN
