# Supplementary material for: Survival outcomes post percutaneous coronary intervention: Why the hype about stent type? Lessons from a healthcare system in India
Source: PLoS One. 2018 May 24;13(5):e0196830. doi: 10.1371/journal.pone.0196830 (PMC5967815; doi:10.1371/journal.pone.0196830)
Supplement: S2 File — (DOC) [file pone.0196830.s002.doc]

**SURVEY-FORM**

**Survey start time:_________________________________Subject ID ______________________________________________________________**

**Name__________________________________________________________________________________________________Age____ Sex__________M/F**

**Homeaddress___________________________________________________________________________________________________________________________________________________________________________________________________________ _Village______________District___________**

**Date of PTCA___________________________ Hospital where PTCA done_______________________________________________________**

**Dt of 1^st^ Interview________2^nd^ ______________3^rd^ _____________4^th^ ______________5^th^____________6^th^_________________**

**Site ID: Mumbai, Out of Mumbai**

**Are you employed_____ If yes do you work on fulltime/part time you work**

**If no.why don't you work___________________________________**

**Do you have diabetes ____________ Last blood sugar level_____**

**What was your last fasting blood sugar level ____**

**Last post prandial bloodsugar ___________**

**What was your HbA1c level:Normal 6.5% < Abnormal _______**

**Do you have Blood pressure(Hypertension)?Hypotension:If BP yes Value of BP_______________**

**If BP yes for how many months____________________________________**

**Alcoholic drink in the last 30 days :Never/Stopped after operation/daily/rarely/**

**Tobacco smoking status____________ ____Cigarette _____________ Bidi___________________Chew tobacco_________________**

**If current smoker frequency bidi per day___________If current smoker frequency cigarette per day_____**

**If current smoker frequency chewtobacco____________**

**Any substance abuse___________________________________**

**Family history of CAD _________________**

**Current Drug use**

**Drug Asprin________ _____Drug Asprin dose ________ Drug Asprin Frequency___________**

**Drug Clopidogrel_____Drug Clopidogrel dose___ Drug Clopidogrel frequency______________**

**Drug Ticlopidine____Drug Ticlopidine dose____Drug Ticlopidinefrequency________________**

**Drug Prasugrel_____ Drug Prasugrel dose______Drug Prasugrel frequency_________________**

**Antihypertensive drugs______ Atenolol/others specify______statin**

**OHA- YES/NO Metformin__ Gliptine__ Pioglitazone__**

**Insulin______**

**Antihypotensive___________ Antianginaal_________**

**Adherence**

**1. Do you sometimes forget to take your heart  pills?**

**2. Over the past two weeks, were there any days when you did not take your heart medicine?**

**3. Have you ever cut back or stopped taking your medication without telling your doctor because you felt worse when you took it?**

**4. When you travel or leave home, do you sometimes forget to bring along your medications?**

**5. Did you take your  medicine yesterday?**

**6. When you feel like alright, do you sometimes stop taking your medicine?**

**7. Taking medication everyday is a real inconvenience for some people. Do you ever feel hassled about sticking to your treatment plan?**

**8. How often do you have difficulty remembering to take all your  medication?**

**Side effects**

**Dyspepsia stomach pain: 0,1,2,3 _Feeling dizzy or lightheaded/headaches:0,1,2,3**

**Nausea or vomiting/diarrhea:01,2,3 Difficulty in breathing,chest tightness:0,1,2,3**

**Difficulty falling or staying asleep:0,1,2,3 Skin rashes/deep purple /bluebruises:0,1,2,3**

**Seizures,depression,anxiety:0,1,2,3Difficultyin micturition:0,1,2,3Arthralgias/backpain:0,1,2,3**

**Followup**

**Have you had a lipid profile done:Yes/No.Have you had chest pain after the procedure:Yes/No**

**If yes what is the degree of chest pain (scale of_(1 to 10) _____**

**How long after the procedure (in days)____**

**After how many days of procedure (indays)______**

**Do you have breathlessness:1,2,3,4,5,6,7,8,9,10 How long after the procedure(in mths)_______**

**Have you ever been hospitalized again after your PTCA_______**

**Have you undergone a repeat procedure__________**

**Have you been adviced for repeat procedure________Which type of vessel_______________________________________**

**What procedure___________________Date of repeat procedure__________________________**

**After how long the 1^st^ procedure was this procedure repeated(in days______________________**

**After how many days of PTCA did patient died ( in days)_______________________________**

**Cardiovascular related illnesses over the past 12 months________________________________**

**Is patient dead_____ When did the patient die____**

**After how many months of surgery did the patient die_____**

**Where did the patient die:Home/Hospital/WaytoHospital/Any other place___________**

**What do you feel the patient died on account off_____**

**Did the patient have chest pain before his death_______________________________________**
