# Supplementary material for: Survival outcomes post percutaneous coronary intervention: Why the hype about stent type? Lessons from a healthcare system in India
Source: PLoS One. 2018 May 24;13(5):e0196830. doi: 10.1371/journal.pone.0196830 (PMC5967815; doi:10.1371/journal.pone.0196830)
Supplement: S3 File — (PDF) [file pone.0196830.s003.pdf]

मरीज का सर्वे करने के बारे मे पुछे जाने वाले सवाल.

१)मरीज नोकरी करता आहे, अथवा अन्य कौनसा व्यवसाय है
२)मरीज को डायबेटीस है कया, उसका तपास किमान ....जदा.....
३)मरीज का HbA1C कितना है........ ....
४)मरीज का बी.पी कितना है.......
५)मरीज पिछले २० दिनोमे शराब पिता था कया.
६)मरीज बीडी, सिगरेट, तंबाखू लेता है का
७)मरीज बीडी,सिगरेट,तंबाखू अलावा अन्य वेसन करता है कया
८)मरीज के कुटूंब मे किसीको ऱ्हदयरोगकी बिमारी है कया
९)कुटूंब मे ऱ्हदयरोगकी बिमारी से मृत्यू हुआ है कया
१०)मरीज कौन कौनसी दवाईंया लेता आहे उनके नाम
११)मरीज को दवाई नजिक के दुकान मे मिलती है या नाही
१२)मरीज को दवाई खरीद ने को परवडता है कया
१३)दवाईकी दुकान नजिक या दूर पर है कया
१४)सरकारने दवाई दे दि तो मरीज ले सकता है कया
१५)सरकारके पास दवाई उपलब्ध हो जाने पर मरीजको एसएमएस/फोन करके बताया जाएगा.
१६)सरकारकी ओर से दवाई दि जानेपर उसका स्टॉक खतम हो जानेपर एसएमएस/फोन करके राजीवगांधी आरोग्य योजना को बताए.
१७)पिछले एक महिनेमे मरीज के प्रकृती मे (पेट दुखना, शिर दुखाना, उल्टी, सीनेमे दर्द, सास लेनेमे तकलीफ, नीद न लगना, बदनपर फोडी आना, पिशाब मे से खून जाना) ऐसा हूआ कया.
१८)चक्कर आना
१९)पिशाब करते समय तकलीफ हुई थी कया.
२०)पिट दुखना, संधीवात होना.
२१)मरीज ने चरबीका तपास किया है कया
२२)एन्जीयोप्लास्टी होनेपर शिनेमे दुखता था कया, दुखता तो दर्द एन्जीयोप्लास्टी के बाद कितने महिने था, और बहूत दुखता या कौसे.
२३)मरीजने दवाई बंद की थी कया, बंद की तो कितने दिनो के लिए एन्जीयोप्लास्टी के बाद बद कि थी
२४)मरीज को धाप लगती है कया, एन्जीयोप्लास्टी के बाद कितने दिन/महीने
२५)मरीज की एन्जीयोप्लाटी होने के बाद दुबारा हॉस्पीटल मे भरती किया था कया
