# Supplementary material for: Survival outcomes post percutaneous coronary intervention: Why the hype about stent type? Lessons from a healthcare system in India
Source: PLoS One. 2018 May 24;13(5):e0196830. doi: 10.1371/journal.pone.0196830 (PMC5967815; doi:10.1371/journal.pone.0196830)
Supplement: S4 File — (PDF) [file pone.0196830.s004.pdf]

२६)मरीज की दुबारा एन्जीयोप्लास्टी की थी कया, अथवा दुबारा एन्जीयोप्लास्टी करनेकी सल्ला दिई गई थी कया.

२७)मरीज की कौनसी एन्जीयोप्लास्टी की थी, तारीख ....

२८) पिछले १२ महिनो मे मरीज की बायपास/पीटीसीए कितनी बार हो गई

२९) मरीज किस तारीख को मृत्यू हो गया ....

३०) एन्जीयोप्लास्टी के बाद कितने दिनोके बाद मृत्यू हो गया (दिन)...

३१)मरीज की मृत्यू कहा हो गई

३२)मरीज किस कारण मृत्यू हो गया

३३)मरीजके सिनेमे मृत्यू के पहिले दर्द हूआ कया

३४)मरीज की एन्जीयोप्लास्टी हो जाने के बाद आनंदी है या नाही
