# Supplementary material for: Survival outcomes post percutaneous coronary intervention: Why the hype about stent type? Lessons from a healthcare system in India
Source: PLoS One. 2018 May 24;13(5):e0196830. doi: 10.1371/journal.pone.0196830 (PMC5967815; doi:10.1371/journal.pone.0196830)
Supplement: S5 File — (PDF) [file pone.0196830.s005.pdf]

१) रुग्ण नोकरी करीत आहे का, नसल्यास व्यवसाय काय आहे.

२) रुग्णास डायबेटीस आहे का, असल्यास शेवटी रक्तसाखरेची तपासणी किती दिवसापुर्वी केली आहे.

रक्तसाखर तपासणी ....

३) रुग्णाचे HbA1c किती आहे.

४) रुग्णाचे Blood Pressure (HT) किती आहे, किती दिवसांपासून आहे.

Blood Pressure ____________

५) रुग्ण मागील ३० दिवसांपुर्वी दारु पित होता का

६) रुग्ण वीडी, सिगरेट, तंबाखू घेतो का

७)रुग्णाला इतर कोणते वेसन आहे का

८) कुटूंबात ऱ्हदयरोगाच्या आजाराने कोण पडीत आहे का

९)कुटूंबात ऱ्हदयरोगाच्या आजाराने मृत्यू झाले आहे का

१०)रुग्ण कोण कोणती औषधे घेत आहे त्याची नावे

११)रुग्णास औषधे जवळच्या दूकानात विकत मिळतात का

१२)रुग्णास औषधे विकत घेणे परवडते का

१३)औषधाचे दुकान जवळ किंवा लांब आहे का

१४)शासनाने औषधे पुरवठा केला तर रुग्ण ती घेऊ शकेल का

१५)शासनाकडे औषधे उपलब्ध झाल्यावर रुग्णास एसएमएस/फोन करुन कळविले जाईल.

१६)रुग्णाचा औषधाचा साठा संपत आल्यावर रुग्णाने एसएमएस/फोन करुन राजीवगांधी आरोग्य योजना यांना कळवावे.

१७)मागील एक महिन्यात आपल्या प्रकृतीत (पोट दूखी, डोके दूखणे, उल्टी,छातीत जड लागणे,स्वशोस्वास घेताना कठीण वाटणे, झोप न येणे, अंगावर दाग /फोडी येणे, लघवीतून रक्त जाणे) विचारणे

१८)चक्कर येणे

१९)लगवी करताना त्रास होणे.

२०)पाट दूखणे, संधीवात आहे का

२१)रुग्णाने चरबीचा तपास केले आहे का

२२)शस्त्रक्रियानंतर छातीत दुखत होते का, दुखत असल्यास त्याचे मोजमाप(१ ते १०), शस्त्रक्रियानंतर किती महीने

२३)रुग्णाने औषधे बंद केली का किती दिवस, बंद केलीत तर किती दिवसांसाठी, शस्त्रक्रिया झाल्यानंतर किती दिवसांसाठी

२४)रुग्णास धाप लागते का शस्त्रक्रियानंतर किती दिवस/महीने

२५)रुग्णाची शस्त्रक्रिया झाल्यानंतर पुन्हा रुग्णालयात दाखल झाला का

२६)रुग्णाची दुसऱ्या वेळेस शस्त्रक्रिया केली का, रुग्णास पुन्हा शस्त्रक्रिया करण्यासाठी सल्ला दिला होता का, ऱ्हदयाच्या कोणत्या रक्तवाहीनीची (एन्जीयोप्लाटी) केली

२७)रुग्णाची कोणती शस्त्रक्रिया केली शस्त्रक्रियाचा दिनांक

पहील्या एन्जीयोप्लास्टी नंतर किती दिवसांनी दुसरी एन्जीयोप्लास्टी केली ( )

एन्जीयोप्लास्टी झाल्यानंतर किती दिवसांनी रुग्ण मरण पावला ( )

२८)मागील १२ महीन्यात रुग्णावर कार्डीयोवासकूलर /पीटीसीए संदर्भातील शस्त्रक्रिया किती वेळा झाली आहे.

२९)रुग्णाचा मृत्यू केव्हा झाला (दिनांक)

३०)शस्त्रक्रियानंतर किती दिवसांनी मृत्यू झाला (दिवस)

३१)रुग्ण कोठे मृत्यू पावला

३२)कशामुळे रुग्ण मृत्यू झाला

३३)रुग्णाच्या छातीत मृत्यूपुर्वी दूखत होते का

३४) रुग्णाची शस्त्रक्रिया झाल्यानंतर आनंदी आहे की नाही

३५) रुग्णाला कुटूंबाने दिलेल्या देखभालवर समाधानी आहे का

३६) रुग्णाच्या कुटूंबाकडून रुग्णाची काळजी/देखभाल घेतली जात आहे का (कोणत्याप्रकारे देखभाल करतात)

३७) रुग्ण इतर कोणाकडून मदत मिळालेल्यांवर समाधानी आहे का
