# Supplementary material for: Survival outcomes post percutaneous coronary intervention: Why the hype about stent type? Lessons from a healthcare system in India
Source: PLoS One. 2018 May 24;13(5):e0196830. doi: 10.1371/journal.pone.0196830 (PMC5967815; doi:10.1371/journal.pone.0196830)
Supplement: S7 File — (PDF) [file pone.0196830.s007.pdf]

Survey table/form:

```
        CHOICES_AGE = [(i,i) for i in range(100)]
        CHOICES_SEX = [('1','Female'),('2','Male')]
        CHOICES_NR = [('0','Yes'),('1','No')]
        CHOICES_Interviewee = [('1','Self'),('2','Relative')]
        CHOICES_EDUCATION =[('1','Illiterate'),('2','Literate
without formal education'),('3','Below primary'),('4','Primary'),
('5','Middle'),('6','Secondary/ Class-10/ Matric'),('7','Hr. Sec/
Sec/ Class XII/ Pre-Univ'),('8','Graduate and Above'),
('9','Vocational'),('88','Other')]
        CHOICES_MARITAL=[('1','Never Married'),('2','Married'),
('3','Remarried'),('4','Widow(er)'),('5','Divorced'),
('6','Seperated'),('98','Refused to answer')]
        CHOICES_YN = [('1','Yes'),('2','No')]
        CHOICES_YNK = [('1','Yes'),('2','No'),('99','Do Not Know')]
        CHOICES_Work = [('1','Full Time'),('2','Part Time Basis')]
        CHOICES_NoWork = [('1','Don\'t want to work'),('2','Lost
job'),('3','Too sick to carry on working'),('4','Don\'t need to
work'),('5','Never worked'),('88','Others'),('98','N / A')]
        CHOICES_ALCOHOL = [('1','Never'),('2','Once'),('3','2-3
times in a month?'),('4','Once or twice a week'),('5','3-4 times a
week'),('6','Nearly every day'),('7','Daily')]
        CHOICES_SMOKE = [('1','Current smoker (last 6 months)'),
('2','Past smoker (6 months ago)'),('3','Non-smoker')]
        CHOICES_SYMPTOM = [('0','I do not have this symptom'),
('1','It doesn\'t bother me'),('2','It bothers me a little'),
('3','It bothers me a lot')]
        CHOICES_LOCATION = [('1','Home'),('2','On way to
hospital'),('3','Transferring from one hospital to other'),('4','Any
OtherAt Hospital'),('99','Not Applicable')]
        CHOICES_LIKERT = [('1','Strongly Disagree'),
('2','DisAgree'),('3','Neutral'),('4','Agree'),('5','Strongly
Agree')]
        CHOICES_LIKERTS = [('1','Very Satisfied'),('2','Not quite
satisfied'),('3','Neutral'),('4','Satisfied'),('5','Very
satisfied')]
        CHOICES_PHQ = [('1','Not at all'),('2','Several Days'),
('3','More than half of the day'),('4','Nearly every day')]



        Subject_ID =  Text_String('Subject ID',  max_length=30,
primary_key=True)
        Name=  Text_String('Name', max_length=30)
        Age=  Numerals_Integers('Age', choices=CHOICES_AGE)
        Sex=  Text_String('Sex', choices=CHOICES_SEX,max_length=30)
        Address_Home=  Text_String('Address Home', max_length=100)
        Village=  Text_String('Village', max_length=30)
        District=  Text_String('District', max_length=30)
        Dt_PTCA=  DateField('Date of PTCA')
        Dt_Interview=  DateField('Date of  interview')
        Dt_Second_Interview= DateField('Date of second
interview',blank=True,null=True)
```

```
        Hospital=  Text_String('Hospital where PTCA done', max_length=30)
        Site_ID=  Text_String('Site ID',choices=[('Mumbai','Mumbai'),('Out of Mumbai', 'Out of Mumbai')], max_length=30)
        Interviewee =  Text_String('Who is answering the questions?       ', choices=CHOICES_Interviewee,max_length=30)
        Consent=  Text_String('Informed Consent obtained',choices=CHOICES_YN, max_length=30)
        Telephone1=  Text_String('Telephone number', max_length=30)
        Telephone2=  Text_String('Alternative phone number ', max_length=30)
        Availability=  DateTimeField('When available?      ', max_length=30)
        Non_Responders =  Text_String('Non responders', choices=CHOICES_NR,max_length=30)
        Contact_times=  Numerals_Integers('Number of times to contact', max_length=30)
        Interviewer=  Text_String('Research Interviewer', max_length=30)
        Data_Entry=  Text_String('Data Entry Operator', max_length=30)
        StartTime=  TimeField('Survey Start Time  ')
        Education_level=  Text_String('What is your highest educational level?', choices=CHOICES_EDUCATION,max_length=30)
        Marital_status=  Text_String('What is your current marital status?',choices=CHOICES_MARITAL, max_length=30)
        Livewith_BYS=  True_False('By your self')
        Livewith_Spouse=  True_False('Spouse')
        Livewith_Parents=  True_False('Parents')
        Livewith_Siblings=  True_False('Siblings')
        Livewith_Children=  True_False('Children')
        Livewith_Relatives=  True_False('Relatives')
        Livewith_Frnd=  True_False('Friends/Roomates')
        Employed_status=  Text_String(' Are You employed?        ',choices=CHOICES_YN, max_length=30)
        Work_Fulltime=  Text_String('If yes do you work on full time or part time basis?',choices=CHOICES_Work,max_length=30, blank=True)
        NoWork_reason=  Text_String('If no, why don\'t you work?',choices=CHOICES_NoWork, max_length=30, blank=True)
        Land=  Text_String('Land',choices=CHOICES_YNK, max_length=5)
        House=  Text_String('House',choices=CHOICES_YNK, max_length=5)
        Car=  Text_String('Car    ',choices=CHOICES_YNK, max_length=5)
        TV=  Text_String('TV',choices=CHOICES_YNK, max_length=5)
        Two_wheeler=  Text_String('Motorbike / Scooter ',choices=CHOICES_YNK, max_length=5)
        Fridge=  Text_String('Refrigerator',choices=CHOICES_YNK, max_length=5)
        AC=  Text_String('AC',choices=CHOICES_YNK, max_length=5)
        Computer=  Text_String('Computer /
```

```
printer ',choices=CHOICES_YNK, max_length=5)
        Washing_Machine=  Text_String('Washing
machine ',choices=CHOICES_YNK, max_length=5)
        AirCooler=
Text_String('Aircooler   ',choices=CHOICES_YNK, max_length=5)
        Cycle=  Text_String('Cycle',choices=CHOICES_YNK,
max_length=5)
        Diabetes=  Text_String('Do you have
Diabetes?',choices=CHOICES_YNK, max_length=30)
        Blood_sugar=  Numerals_Integers('last blood sugar level ?',
max_length=30 , null=True, blank=True)
        Fasting_Blood_sugar=  Numerals_Integers('What was your last
fasting blood sugar level? ', max_length=30,null=True,blank=True)
        Postprandial_blood_sugar=  Numerals_Integers('last
postprandial blood sugar level?', max_length=30,null=True,
blank=True)
        HbA1c=  Decimal_Number('What was your HbA1c level?',
max_digits=5, decimal_places=2)
        Hypertension=  Text_String('Do you have  Blood
pressure(hypertension)?',choices=CHOICES_YNK, max_length=30)
        Value_Hypertension=  Text_String('If BP yes, Value of
BP?     ', max_length=30, blank=True)
 Month_Hypertension=  Decimal_Number('If BP yes, for how many
Months?', max_digits=5, decimal_places=2)
        Alcohol=  Text_String('Alcoholic drink in the last 30
days ?',choices=CHOICES_ALCOHOL, max_length=30)
        Tobacco_status=  Text_String('Tobacco smoking
status?',choices=CHOICES_SMOKE, max_length=30)
        Smoke_Cigar=  True_False('Cigarette')
        Smoke_Beedi=  True_False('Beedi')
        Smoke_Tobacco=  True_False('Chew Tobacco')
        Beedis=  Text_String('If current smoker, frequency- beedis
per day ',choices=CHOICES_ALCOHOL, max_length=30,blank=True)
        Cigar=  Text_String('If current smoker, frequency-
cigarettes per day',choices=CHOICES_ALCOHOL,
max_length=30,blank=True)
        Chew_tobacco=  Text_String('If Chew tobacco, frequency-
others per day',choices=CHOICES_ALCOHOL, max_length=30,blank=True)
        Substance_abuse=  Text_String('Any other substance abuse',
choices=CHOICES_YN,max_length=30)
        CAD_History=  Text_String('Family History of
CAD',choices=CHOICES_YN, max_length=30)
        CAD_Report=  Text_String('If Yes, Which Family Member and
at what age?', max_length=30,blank=True)
        Sudden_death_History=  Text_String('History of Sudden death
in the family', choices=CHOICES_YN,max_length=30)
        Aspirin=  Text_String('Drug Asprin',choices=CHOICES_YN,
max_length=30)
        Aspirin_dose=  Text_String('Drug Asprin Dose',choices=[('N/
A','N/A'),('50','50'),('75','75'),('150','150'),('325','325')],
max_length=30)
        Asprin_Freq=  Text_String('Drug Asprin
Frequency',choices=[('1','Once a day'),('2','Twice a day')],
max_length=30,blank=True)
```

```
        Clopidogrel =  Text_String('Drug
Clopidogrel',choices=CHOICES_YN, max_length=30)
        Clopidogrel_dose=  Text_String('Drug Clopidogrel
Dose',choices=[('N/A','N/A'),('75','75'),('150','150')],
max_length=30)
        Clopidogrel_Freq=  Text_String('Drug Clopidogrel
Frequency       ',choices=[('1','Once a day'),('2','Twice a day')],
max_length=30,blank=True)
        Ticlopidine =  Text_String('Drug
Ticlopidine',choices=CHOICES_YN, max_length=30)
        Ticlopidine_Dose =  Text_String('Drug Ticlopidine
Dose    ', max_length=30,blank=True)
        Ticlopidine_Freq=  Text_String('Drug Ticlopidine
Frequency',choices=[('1','Once a day'),('2','Twice a day')],
max_length=30,blank=True)
        Prasugrel =  Text_String('Drug
Prasugrel',choices=CHOICES_YN, max_length=30)
        Prasugrel_Dose=  Text_String('Drug Prasugrel Dose',
max_length=30, blank=True)
        Prasugrel_Freq=  Text_String('Drug Prasugrel
Frequency',choices=[('1','Once a day'),('2','Twice a day')],
max_length=30, blank=True)
        Antihypertension =  Text_String('Antihypertension
drugs',choices=CHOICES_YN, max_length=30)
        Atenol_or_other=  Text_String(' Atenolol/others specify',
max_length=30)
        OHA_Oral=  Text_String('OHA_Oral',
choices=CHOICES_YN,max_length=30)
        Insulin=  Numerals_Integers(' Insulin', max_length=30)
        Drugs_availibility=  Text_String('Prescribed drugs are
easily available at the pharmacy',choices=CHOICES_YN, max_length=30)
        Medicine_afford=  Text_String('If yes, are you able to
afford the required medicine?',choices=CHOICES_YN, max_length=30,
blank=True)
        Pharmacy_close=  Text_String('Is the pharmacy/ dispensary
close to your home?',choices=CHOICES_YN, max_length=30)
        Designated_pharmacy=  Text_String('designated  pharmacy
centers',choices=CHOICES_YN, max_length=30)
        Notification_Type=  Text_String(' informed through sms or
an automated call',choices=CHOICES_YN,max_length=30)
        Send_notification=  Text_String('send an SMS or call  or
when your supply is finishing ?',choices=CHOICES_YN, max_length=30)
        Dyspepsia=  Text_String('Dyspepsia, stomach
pain',choices=CHOICES_SYMPTOM, max_length=30)
        Dizzy=  Text_String('Feeling dizzy or lightheaded/
headaches',choices=CHOICES_SYMPTOM, max_length=30)
        Nausea=  Text_String('Nausea or vomiting/
diarrhoea       ',choices=CHOICES_SYMPTOM, max_length=30)
        Breathing=  Text_String('Difficulty in breathing, chest
tightness',choices=CHOICES_SYMPTOM, max_length=30)
        Sleep=  Text_String('Difficulty falling or staying
asleep  ',choices=CHOICES_SYMPTOM, max_length=30)
        Skin=  Text_String('Skin rashes/deep purple or blue
bruises ',choices=CHOICES_SYMPTOM, max_length=30)
```

```
        Depression=  Text_String('Seizures, depression,
anxiety ',choices=CHOICES_SYMPTOM, max_length=30)
        Micturition=  Text_String('Difficulty in
micturition',choices=CHOICES_SYMPTOM, max_length=30)
        Arthralgia =  Text_String('Arthralgias/back
pain',choices=CHOICES_SYMPTOM, max_length=30)
        Lipid=  Text_String('Have you had a Lipid profile
done?',choices=CHOICES_YNK, max_length=30)
        Chest_Pain=  Text_String('Have you had chest pain after the
procedure?',choices=CHOICES_YNK, max_length=30)
        Degree_Chest_Pain=  Numerals_Integers(' if Yes, What is
degree of chest pain (scale of 1 to 10)',choices=[(i,i) for i in
range(0,11)])
        Long_after_procedure_Chest_Pain=  Decimal_Number('How long
after procedure (in months)?',max_digits=5, decimal_places=2)
        Stop_Medication=  Text_String('Did you stop medications and
for how many days?',choices=CHOICES_YNK, max_length=30)
        Days=  Numerals_Integers('If Yes, for how many days?',
max_length=30)
        Days_after_procedure_Stop_Medication=
Numerals_Integers('After how many days of procedure? (In
Days)',max_length=30)
        Breathlessness=  Text_String('Do you have breathlessness?',
choices=CHOICES_YNK, max_length=30)
        Long_after_procedure=  Decimal_Number('How long after the
procedure (In Months)', max_digits=5, decimal_places=2)
        Hospitalized_after_PCTA=  Text_String('Have you ever been
hospitalized again after your PTCA ?',choices=CHOICES_YN,
max_length=30)
        Repeat_procedure=  Text_String('Have you undergone a repeat
procedure?',choices=[('1','Yes'),('2','No')], max_length=30)
        Adviced_repeat_procedure= Text_String('Have you been
advices for repeat procedure?',choices=[('1','Yes'),('2','No'),
('3','N/A')], max_length=30)
        Type_vessel= Text_String('Which type of vessel?',
choices=[('1','Same Vessel'),('2','Different Vessel'),('3', 'Dont
Know'),('4','N/A'),], max_length=30)
        What_procedure= Text_String('What
Procedure?',choices=[('1','PTCA'),('2','Bypass'),('3','N/A')],
max_length=30)
        DT_repeat_procedure= DateField('Date of repeat
procedure?',blank=True,null=True)#new added 12 Nov
        Long_repeat_procedure= Numerals_Integers('After how long of
first procedure was this procedure repeated? (in
days)',max_length=30)#new added 12 Nov
        Die_Days_PTCA= Numerals_Integers('After how many DAYS of
PTCA did patient died? (in days)',max_length=30)
        CVD_illness=  Text_String('cardiovascular related illnesses
over in the past 12 months?       ', max_length=30)
        Is_Dead= Text_String('Is patient
dead?',choices=[('1','No'),('2','Yes')],default='1',max_length=30)
        DT_death=  DateField('When did the patient
died?',blank=True,null=True)
        Month_death=  Decimal_Number('After how many months of
```

```
surgery did the patient die?      ', max_digits=5, decimal_places=2)
        Location_death=  Text_String('Where did the patient 
die?',choices=CHOICES_LOCATION,max_length=30)
        Reason_death=  Text_String('What  do you feel the patient 
died on account of?      ', max_length=30)
        Pain_Death=  Text_String('Did the patient have chest pain 
before his death?        ',choices=CHOICES_YNK, max_length=30)
        Medication_necessity=  Text_String('Medications are 
necessary once a stent has been deployed?',choices=CHOICES_YNK, 
max_length=30)
        Medication_stop=  Text_String('Medications should be 
stopped?',choices=CHOICES_YNK, max_length=30)
        Disease_seriousness=  Text_String('Disease has become less 
serious because of medications?',choices=CHOICES_LIKERT, 
max_length=30)
        Worth_Medication=  Text_String('Medications are not 
worth?',choices=CHOICES_LIKERT, max_length=30)
        PCTA_Medication_Stop=  Text_String('After PTCA Surgery , 
medications can  be stopped?',choices=CHOICES_LIKERT, max_length=30)
        Pleasure=  Text_String('Little interest or pleasure in 
doing things     ', choices=CHOICES_PHQ, max_length=30)
        Depress=  Text_String('Feeling depressed , down or 
hopeless ',choices=CHOICES_PHQ, max_length=30)
        Call_nurse=  Text_String('Would you prefer call from a 
nurse?',choices=CHOICES_YNK, max_length=30)
        Helpline=  Text_String('Would you like to have a 
helpline?',choices=CHOICES_YNK, max_length=30)
        Report_event=  Text_String('report an adverse 
event?',choices=CHOICES_YNK, max_length=30)
        Connectivity=  Text_String('Do you use internet on phone or 
at home?/ Email address  ',choices=CHOICES_YNK, max_length=30)
        Email=  Text_String('Do you have an email 
account? ',choices=CHOICES_YNK, max_length=30)
        Family_support=  Text_String('Do you receive support from 
your family? Please also mention the type of 
support',choices=CHOICES_YN, max_length=30)
        #Support_kind=  Text_String('What kind of support do you 
get from your family?', max_length=30, blank=True)
        S_Psychological =  True_False('Psychological support')
        S_Financial =  True_False('Financial support')
        S_Physical =  True_False('Physical care')
        S_Other =  Text_String('Other (Please Specify)', 
max_length=30, blank=True)
        Support_satisfied=  Text_String('how satisfied are you with 
the overall support?',choices=CHOICES_LIKERTS, max_length=30, 
blank=True)
        Support_outside=  Text_String('Do you receive support from 
outside? If yes, please mention the support 
source',choices=CHOICES_YN, max_length=30)
        SS_Friend =  True_False('Friends')
        SS_NGO =  True_False('NGO')
        SS_CBO =  True_False('Community based organization')
        SS_RBO =  True_False('Religious based organization')
        SS_GF =  True_False('Government facility')
```

```
        SS_WP =  True_False('Workplace programs')
        SS_Other =  Text_String('Other (Please Specify)',
max_length=30, blank=True)
        OutsideSupport=  Text_String('Do you get support from
outside your home? If yes also mention the type of support.',
max_length=30)
        OS_Psychological =  True_False('Psychological support')
        OS_Financial =  True_False('Financial support')
        OS_Physical =  True_False('Physical care')
        OS_Income =  True_False('Income generating activities')
        OS_Nutritional =  True_False('Nutritional support')
        OS_Legal =  True_False('Legal support')
        OS_Other =  Text_String('Other (Please Specify)',
max_length=30, blank=True)
        OutsideSupport_satisfied=  Text_String('In general, how
satisfied are you with the overall support you get from your outside
your home?',choices=CHOICES_LIKERTS, max_length=30, blank=True)
        Time_SurveyEnd=  TimeField('Survey End Time')
```
